# Malten, a new synthetic molecule showing *in vitro* antiproliferative activity against tumour cells and induction of complex DNA structural alterations

**DOI:** 10.1038/sj.bjc.6605745

**Published:** 2010-06-22

**Authors:** S Amatori, I Bagaloni, E Macedi, M Formica, L Giorgi, V Fusi, M Fanelli

**Affiliations:** 1 Molecular Pathology and Oncology Lab. ‘PaoLa’, Department of Biomolecular Sciences, University of Urbino ‘Carlo Bo’, via Arco d’Augusto, 2, 61032 Fano (PU), Italy; 2 Institute of Chemical Sciences, University of Urbino ‘Carlo Bo’, p.zza Rinascimento, 6, 61029 Urbino (PU), Italy

**Keywords:** antineoplastic drug, apoptosis, cell cycle, cytotoxicity, cancer therapy

## Abstract

**Background:**

Hydroxypyrones represent several classes of molecules known for their high synthetic versatility. This family of molecules shows several interesting pharmaceutical activities and is considered as a promising source of new antineoplastic compounds.

**Methods:**

In the quest to identify new potential anticancer agents, a new maltol (3-hydroxy-2-methyl-4-pyrone)-derived molecule, named malten (*N,N*′-bis((3-hydroxy-4-pyron-2-yl)methyl)-*N,N*′-dimethylethylendiamine), has been synthesised and analysed at both biological and molecular levels for its antiproliferative activity in eight tumour cell lines.

**Results:**

Malten exposure led to a dose-dependent reduction in cell survival in all the neoplastic models studied. Sublethal concentrations of malten induce profound cell cycle changes, particularly affecting the S and/or G2-M phases, whereas exposure to lethal doses causes the induction of programmed cell death. The molecular response to malten was also investigated in JURKAT and U937 cells. It showed the modulation of genes having key roles in cell cycle progression and apoptosis. Finally, as part of the effort to clarify the action mechanism, we showed that malten is able to impair DNA electrophoretic mobility and drastically reduce both PCR amplificability and fragmentation susceptibility of DNA.

**Conclusion:**

Taken together, these results show that malten may exert its antiproliferative activity through the induction of complex DNA structural modifications. This evidence, together with the high synthetic versatility of maltol-derived compounds, makes malten an interesting molecular scaffold for the future design of new potential anticancer agents.

Despite increasing advances in modern treatments for tumours, the survival of many patients affected by cancer remains extremely poor. The high possibility of relapse, the absence of specific therapies and resistance to standard chemotherapy represent the main causes of failure in cancer treatment (Souhami and Tobias, 2005). There is an urgent necessity for research focused on the identification and description of new antitumour agents.

Hydroxypyrones and their close congeners, hydroxypyridinones, comprise several classes of molecules characterised by high synthetic versatility. Their affinity for a wide range of metal ions makes them ideal metal chelators in the formulation of therapeutic and diagnostic metallopharmaceuticals. These families of molecules are used, or have good potential, in clinical applications, such as in the improvement of metal ion balance in disorders of metal metabolism, the enhancement of tissue uptake and retention of insulin-enhancing agents, as well as in protection from heavy metal ion toxicity and attenuation of redox metal neurotoxicity (Thompson *et al*, 2006).

Maltol (3-hydroxy-2-methyl-4-pyrone), one of the best known hydroxypyrones, is a natural compound extensively used for its flavour and antioxidant properties (Gralla *et al*, 1969; Bjeldanes and Chew, 1979) as an additive in food, beverage, tobacco, brewing and cosmetics. Although its high bioavailability and favourable toxicity profiles have been known for a long time, maltol was found to perform interesting antineoplastic activities against different cancer cellular models. Reactive oxygen species (ROS) generation and the consequent induction of DNA breaks have been hypothesised as a possible mechanism responsible for this activity (Hironishi *et al*, 1996; Yasumoto *et al*, 2004; Murakami *et al*, 2006a, 2006b).

Furthermore, several maltol-derived compounds have been synthesised and exploited in the formulation of new potential metal-based antitumour drugs that can interfere with DNA (e.g., DNA binding, DNA intercalation and ROS generation), providing interesting results (Jakupec and Keppler, 2004; Barve *et al*, 2009; Kandioller *et al*, 2009). In the belief that these compounds have good potential as anticancer agents, we synthesised new maltol-derived molecules and investigated their biological activity.

In this study, the antineoplastic potential of a selected molecule, malten (*N,N*′-bis((3-hydroxy-4-pyron-2-yl)methyl)-*N,N*′-dimethylethylendiamine), was evaluated for its ability to decrease the survival of eight different tumour cell lines. Subsequently, cell cycle modifications and the induction of programmed cell death were investigated at both biological and molecular levels to clarify the biological response to malten exposure. Finally, the malten action mechanism was investigated in both cellular and cell-free assays, directing attention to the possible cause of changes in DNA structure.

## Materials and methods

### Chemicals and synthesis of malten

All chemicals and compounds used in this study

- maltol,
- *N,N*′-dimethylethylendiamine (DMEDA),
- 3-hydroxy-2-dimethylaminomethyl-4-pyrone (DMAMP), and
- cisplatin (CDDP)

were of the highest quality, commercially available and purchased from Sigma-Aldrich (St Louis, MO, USA). The synthesis of malten (research paper in preparation) was carried out as follows: a protected maltol derivative was synthesised by silylation and allylic bromination of commercial maltol. After removal of the hydroxyl protective group and without purification, the maltol derivative was combined with *N,N*′-dimethylethylenediamine. Malten was then purified by precipitation as perchlorate salt, obtaining an overall yield of 50%.

### Cell cultures, treatments and cell survival estimates

Immortalised promonocytic leukaemia (U937), acute promyelocytic leukaemia (NB4), acute myeloid leukaemia (HL-60), T-cell leukaemia (JURKAT), glioblastoma multiforme (U-373MG), cervix carcinoma (HeLa), malignant pleural mesothelioma (H28) and alveolar rhabdomyosarcoma (RH30) human tumour cell lines were obtained from American Type Culture Collection (ATCC, Rochville, MD, USA).

Cellular populations derived from solid tumours were cultured in DMEM (Cambrex, Walkersville, MD, USA), whereas cells derived from haematopoietic malignancies were grown in RPMI 1640 (Cambrex, Walkersville, MD, USA). Both culture media were supplemented with 10% foetal bovine serum, 1% penicillin–streptomycin and 1% glutamine. All cell lines were grown in a humidified atmosphere at 37°C as previously described (Fanelli *et al*, 2008).

Malten was dissolved at 25 mM in double-distilled water as stock solution, stored at −80°C and subsequently diluted before use. Treatments were carried out at the concentrations reported in the figures and repeated every 24 h. Cellular viability was evaluated by Trypan blue dye exclusion assay 72 h after treatment started. The 50% inhibitory concentration (IC50) was calculated for each cell line as the drug concentration, which resulted in a 50% reduction of viable cells compared with untreated cells.

### Cell cycle analysis and hypodiploid cells estimation

Cell cycle and hypodiploid cells were analysed using the propidium iodide staining procedure as previously reported (Amatori *et al*, 2009). Cells were fixed in ice-cold 70% ethanol and stained using a propidium iodide staining solution (50 *μ*g ml^−1^). Cytofluorimetric acquisitions were carried out with a BD FACScalibur flow cytometer (BD Biosciences, San Jose, CA, USA) and sample analysis was carried out with FlowJo 8.6.3 software (Tree Star, Inc., Ashland, OR, USA). Cell cycle percentage values were calculated using a Watson pragmatic model and the significance of changes was evaluated by a Student *t*-test (*P*<0.05).

### DNA laddering assay

About 2 × 10^6^ cells were harvested, washed once with ice-cold PBS and incubated in 2 ml of 1 × TBS, 0.5% Tween20 and 1 mM EDTA for 30 min at 4°C. Cells were centrifuged at 1200 r.p.m. for 5 min at 4°C and incubated with 0.1% SDS solution for 30 min at room temperature, in a final volume of 0.5 ml. DNA was purified using a QIAquick Gel Extraction Kit (Qiagen, Hilden, Germany), following the manufacturer's instructions, separated by 2% agarose gel electrophoresis (AGE) and detected by ethidium bromide staining.

### Quantitative reverse transcriptase(Q-RT)–PCR and western blotting

RNA isolation, real-time RT–PCR assay and subsequent analysis were carried out as previously described (Caprodossi *et al*, 2005). Amplifications were carried out using a PCR array designed to simultaneously analyse the expression levels of 37 different genes in 100-well discs (Corbett Life Science, Sydney, Australia–research paper in preparation). The following genes, known to have key roles in cell cycle regulation, were investigated (primer sequences available on request):

*ABL1, ATM, ATR, BAX, BCL2, BIRC5 (survivin), BRCA1, BRCA2, CCNB1 (cyclin B1), CCNB2 (cyclin B2), CCNE1 (cyclin E1), CDC16, CDC2, CDC20, CDC34, CDK2, CDK4, CDK6, CDK7, CDK8, CDKN1A (p21), CDKN1B (p27), CDKN2A (p14-p16), CDKN2B (p15), CDKN3 (KAP), CHEK1, CHEK2, CUL3, E2F4, GADD45A, KNTC1, MCM2, MKI67 (Ki-67), PCNA, RPA3, TP53, UBE1*.

Primers were designed using Primer Express software (PE Applied Biosystem, Foster City, CA, USA) and PCR assays were applied in a Rotor-Gene 6000 robocycler (Corbett Life Science, Sydney, Australia). The expression of different transcripts was normalised with GAPDH expression levels, and calculated as fold induction with respect to untreated cells. Filtering of results was carried out as follows: genes were considered regulated when their change was greater than ±2.5-fold.

Western blotting analyses were performed as previously described (Fanelli *et al*, 2008). Anti-p15 (#4822), anti-p21 (DCS60–#2946), anti-CDK6 (DCS83–#3136) and anti-PCNA (PC10–#2586) antibodies were purchased from Cell Signalling Technology (Beverly, MA, USA); anti-p16 (H156–sc759) and anti-p53 (pDO-1–sc126) antibodies were purchased from Santa Cruz Biotechnology (Santa Cruz, CA, USA); the anti-*α* tubulin (Clone DM1A–T9026) antibody was purchased from Sigma Aldrich (St Louis, MO, USA); and anti-BRCA1 (Ab-1 #OP92) was purchased from Calbiochem (San Diego, CA, USA).

### DNA electrophoretic mobility assay

A volume of 100–500 ng of circular pLL3.7 plasmid DNA (resuspended in 1 × TE) was incubated with the compounds tested in 20 *μ*l of 10 mM Tris-HCl (pH 7.4) buffer (37°C), at the reported concentrations and time points. After incubation, DNA was separated by 0.8% AGE and then stained by ethidium bromide. Ascorbic acid and CuSO_4_ at equimolar concentrations (50 nM) were used as a control of plasmid linearisation induced by DNA breaks (Gaetke and Chow, 2003).

Alternatively, linear pLL3.7 plasmid DNA, previously incubated with malten or CDDP for 2 h at 37°C under conditions already described, was partially fragmented through mild sonication (once × 10 s at 20 W–Labsonic L sonicator–B. Braun Biotech International, Melsungen, Germany) and separated by AGE.

DNA electrophoretic mobility assay of genomic DNA was carried out as follows: U937 cells, treated with malten for 8 h at the concentrations reported, were harvested, washed with ice-cold 1 × PBS, resuspended in a solution containing 1 × PBS, 0.1% SDS, 10 *μ*g/ml RNase A and incubated overnight at 4°C. Genomic DNA was separated by AGE and detected using ethidium bromide staining.

### PCR inhibition assay

A volume of 500 ng of pLL3.7 plasmid DNA (in 1 × TE), incubated with 4 mM malten (2 h at 37°C) under conditions already described, was diluted to 0.25 pg/*μ*l with double-distilled water and amplified (2 *μ*l) by real-time Q-PCR using the following sets of primers—characterised by a common reverse primer and able to amplify the same plasmid region producing amplicons of different length (121, 301 and 622 bp):

pLLF1: 5′-AATACCGCGCCACATAGCAG–3′,

pLLF2: 5′-GTTGTCAGAAGTAAGTTGGCCGC–3′,

pLLF3: 5′–GCTGCAATGATACCGCGAGAC–3′ and

pLLR: 5′-GTGCACGAGTGGGTTACATCG–3′.

Q-PCR experiments and subsequent analysis were carried out as previously described (Caprodossi *et al*, 2005).

## Results

### Malten synthesis and antiproliferative effects against tumour cell lines

**Figure 1:**
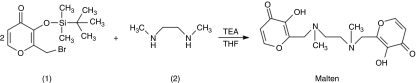
Scheme of malten synthesis. (1) maltol derivative; (2) *N,N*′-dimethylethylenediamine.

The new maltol-derived molecule malten, prepared as shown in Figure 1 (described in Material and Methods), was obtained after precipitation as a white solid perchlorate salt (L.2HClO_4_), light- and air stable, easy to handle and, more importantly for future applications, soluble in water.

**Figure 2:**
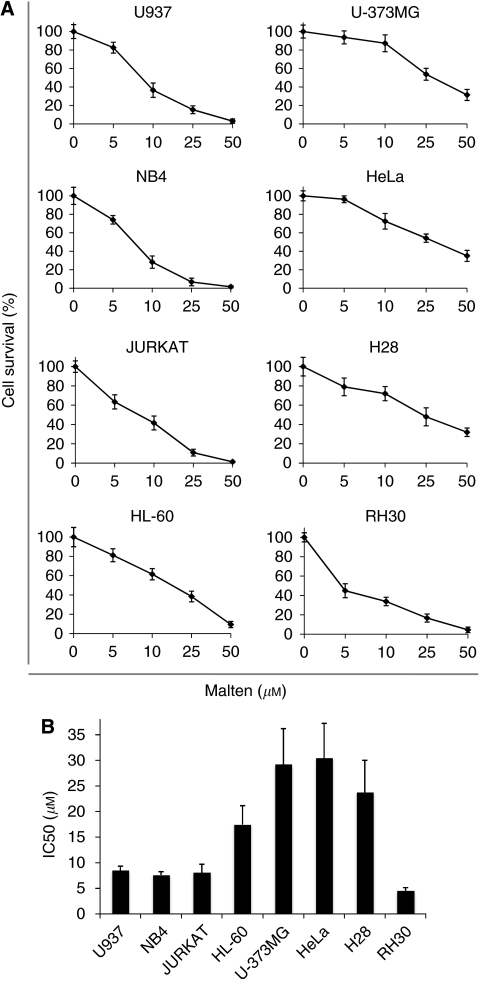
Reduction of cell survival induced by malten treatments in eight different tumour cell lines. Cells were treated for 72 h with the reported concentrations of malten and analysed by Trypan blue dye exclusion assay. The data are reported as mean (±s.d.) resulting from three independent experiments. (**A**) Dose–response experiments. (**B**) IC50.

Different tumour cell lines, comprised of cells derived from both solid and haematopoietic malignancies, were subjected to 72 h of exposure to malten in dose–response experiments to evaluate the biological effects. We found that treatments with malten at concentrations ranging between 5 and 50 *μ*M result in a dose-dependent reduction in cell survival (Figure 2A). Subsequently, IC50 value was calculated for each cell line showing appreciable variations (from 4.54±0.66 *μ*M to 30.47±6.75 *μ*M) and indicating a certain cell-type specificity of malten activity (Figure 2B). Interestingly, RH30 was the most sensitive cellular population suggesting the presence of peculiar molecular features in alveolar rhabdomyosarcoma that could render this cell line more susceptible to malten exposure.

### Analysis of malten-mediated cell cycle perturbations and induction of programmed cell death

**Figure 3:**
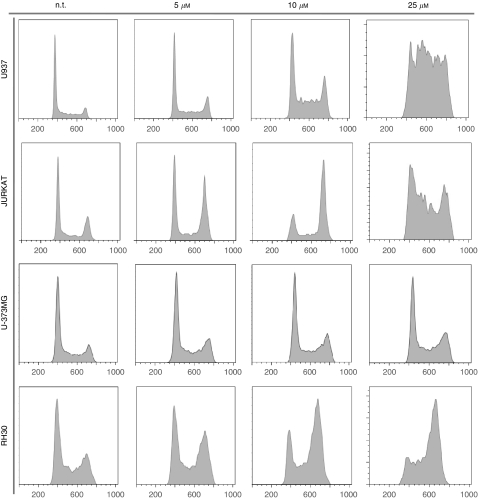
Examples of malten-induced cell cycle perturbation in tumour cells. Cells were exposed for 72 h to the reported concentrations of malten (n.t.=not treated cells). Cell cycle profiles have been evaluated by flow cytometric analysis of propidium iodide-stained cells (see Supplementary Figure S1 for the other cell lines tested).

The biological response to malten was further detailed by analysing the effects on cell cycle progression. Malten treatment of 72 h, at concentrations of 5, 10 and 25 *μ*M, induced cell cycle perturbations that could be monitored in all the cell lines considered in this study. The effects were dose dependent and are characterised by the accumulation of cells mainly in S and/or G2-M phases (Figure 3; Table 1 and Supplementary Figure S1). Interesting differences between the cell lines were also detected. In particular, JURKAT and RH30 cells showed a robust reduction of cells in G1 phase concomitantly with an evident G2-M cell cycle arrest (Figure 3 and Table 1).

**Figure 4:**
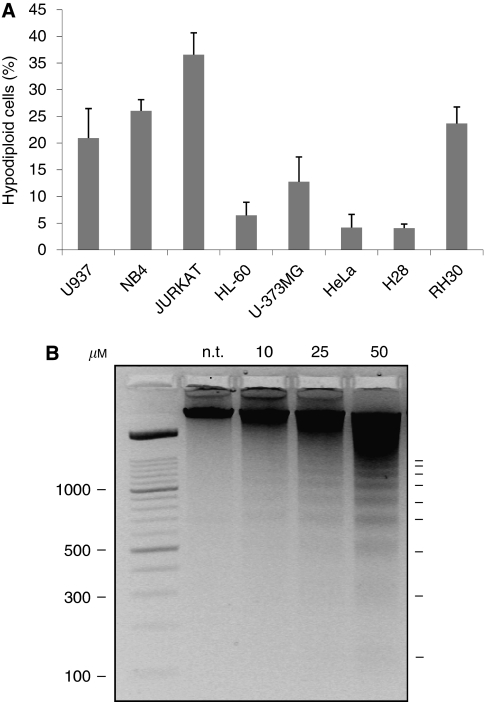
Malten-mediated induction of programmed cell death. (**A**) Percentage of hypodiploid cells induced by malten at the final concentration of 50 *μ*M. Cells were analysed by propidium iodide staining and flow cytometric analysis; hypodiploidy induction was calculated as the difference between the percentages of hypodiploid cells in treated and not treated samples (see Supplementary Figure S2 for cytometric analysis of all the malten doses tested for each tumour cell line). (**B**) Apoptosis-related DNA fragmentation induced by 72 h malten exposure in U937 cells at the final concentration of 10, 25 and 50 *μ*M (n.t.=not treated cells).

Malten-mediated apoptosis was also evaluated, after 72 h of treatment, monitoring an increase of hypodiploid cells (Figure 4A and Supplementary Figure S2), with differences observed between the cell lines tested. Activation of the apoptotic programme was subsequently confirmed by analysing the internucleosomal DNA fragmentation induced by malten exposure in U937 cells, at concentrations of 10, 25 and 50 *μ*M (Figure 4B).

The biological effects mediated by malten treatments were also investigated at the molecular level by analysing the expression of a panel of genes known to regulate cellular proliferation, apoptosis and cell cycle progression.

**Figure 5:**
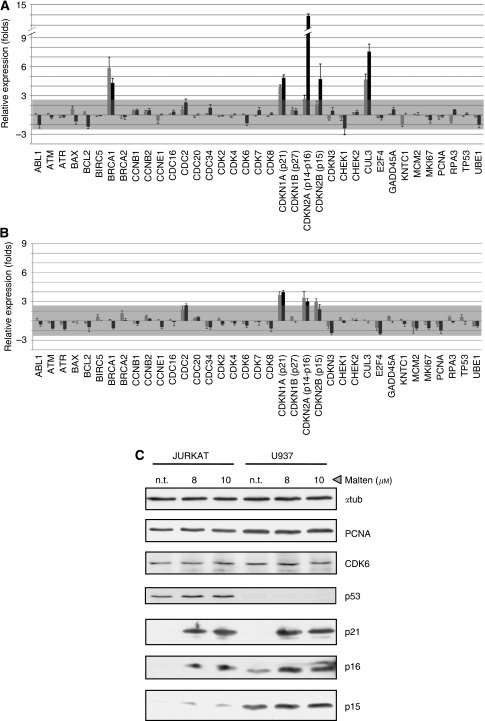
Gene expression modifications induced by malten. JURKAT (**A**) and U937 (**B**) cells were subjected to malten treatments at the concentration of 8 *μ*M (grey bars) and 10 *μ*M (black bars). Transcript abundance of genes involved in cell cycle regulation, cellular replication and apoptotic response was assessed by Q-PCR, normalised with GAPDH expression and evaluated as fold induction with respect to untreated cells. (**C**) Western blot analysis of malten-treated and untreated (n.t.) JURKAT and U937 total cell lysates.

JURKAT and U937 cells were chosen as representative model systems that recapitulate the different biological responses of the cell lines tested. The results obtained by comparing untreated cells with those treated with malten at concentrations of 8 and 10 *μ*M (across the IC50 value) show changes in the mRNA level involving genes having key roles in the control of cell cycle and DNA damage response (Figure 5A and B). In particular, among cyclin-dependent kinase inhibitors (CDKIs), we found the evident upregulation of p21 (CDKN1A), p15 (CDKN2B) and p16 (CDKN2A) in both cell lines and at both malten doses, whereas p27 (CDKN1B) remained unchanged (Figure 5A and B).

The upregulation of BRCA1 and CUL3 at both doses of malten was only observed in JURKAT cells (Figure 5A). Subsequently, genes showing the highest transcriptional modulations were further investigated at protein level. Western blot analysis confirmed the increased expression of p21, p16 and p15 in both cell lines, whereas no changes were monitored for PCNA and CDK6 proteins as they were already monitored at mRNA level (Figure 5C).

P53 is known to be functionally activated prevalently by protein stabilisation, hence its accumulation was monitored. We observed a slight increase in JURKAT cells after malten treatments and, as expected (Decker *et al*, 2003), the complete absence of expression in U937 cells (Figure 5C). The expression level of BRCA1 protein was not detected, probably because of the low sensitivity of the antibody tested.

### Evaluation of DNA structural modifications induced by malten

**Figure 6:**
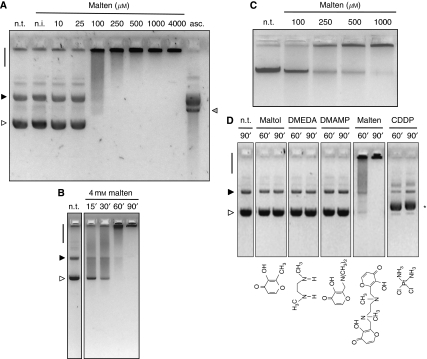

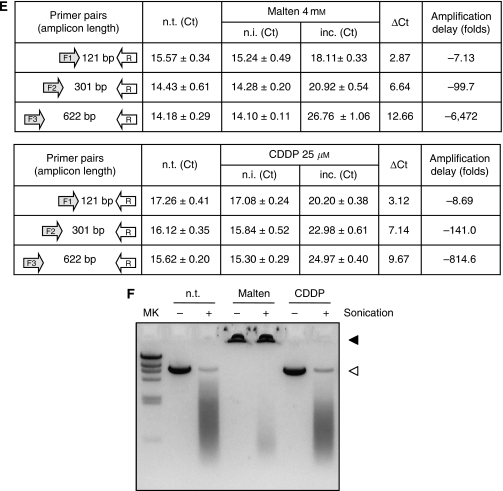
Effects induced by malten on the DNA structure. (**A**) Effects on the electrophoretic migration of plasmid DNA (pLL3.7) were investigated by monitoring the supercoiled (white arrow) and open circular (black arrow) forms abundance, the induction of plasmid linearisation (grey arrow) and the formation of high-molecular-weight DNA complexes (black bar) after 16 h of exposure to different doses of malten. Not treated (n.t.); not incubated (n.i.). Incubation with ascorbic acid (asc.) in the presence of CuSO_4_ for 2 h as control of plasmid linearisation mediated by ROS. (**B**) Effects on the electrophoretic migration of plasmid DNA (pLL3.7) after short times of exposure to 4 mM malten. (n.t.), not treated. (**C**) Genomic DNA modifications induced in U937 cells exposed for 8 h to the reported concentrations (*μ*M) of malten. (**D**) DNA modifying ability of maltol, DMEDA, DMAMP, malten and CDDP at the concentration of 4 mM. The molecular structure of the compounds tested is schematised at the bottom of the figure. ^*^Retardation in the migration of the supercoiled plasmid form. (**E**) PCR inhibition assay. After incubation for 2 h in the presence of 4 mM malten (upper panel) or 25 *μ*M CDDP (lower panel), circular plasmid DNA (pLL3.7) was amplified by Q-PCR using the sets of primers reported in the figure. ΔCt was calculated for each set of primers as the difference between the Ct values of incubated (inc.) and not incubated (n.i.) samples (see Supplementary Figure S3 for treatment of CDDP at the same concentration of malten). (**F**) DNA fragmentation assay. After incubation for 2 h in the presence of malten or CDDP (both at the concentrations of 4 mM), linearised pLL3.7 plasmid DNA was subjected or not to sonication, and separated by AGE. Linearised plasmid DNA (white arrow); high–molecular-weight DNA complexes (black arrow).

As already reported, maltol can damage DNA through ROS production (Murakami *et al*, 2006a, 2006b). We first tested the hypothesis that malten could cause DNA breaks by analysing its effect on circular plasmid DNA by AGE. Dose–response experiments showed that after 16 h of malten exposure, there was neither plasmid linearisation nor evident DNA degradation (Figure 6A). However, we observed that malten is able to modify the electrophoretic mobility of plasmid DNA causing the complete trapping of DNA at the top of the gel at the higher concentrations tested. This phenomenon is strictly dependent on incubation, as shown by the normal pattern of migration of plasmid DNA at the higher malten dose (4 mM) when incubation was omitted (Figure 6A, lane n.i.), indicating that the presence of malten *per se* does not disturb the electrophoretic migration of DNA. DNA complexes with impaired electrophoretic mobility were also found using shorter exposure times and a malten concentration of 4 mM (Figure 6B).

We then investigated malten-induced effects on the genomic DNA of cancer cells. U937 cells were exposed to different doses of malten (from 0.1 to 1 mM) for a relatively short period of time (8 h) to maximise the effect of the compound and avoid any interference from the biological response (e.g., internucleosomal cleavage of DNA related to the apoptotic response). We found that U937 cells exposed to malten show a concentration-dependent reduction of electrophoretic mobility of their genomic DNA (Figure 6C).

Having established that the induction of DNA modifications can also occur in the cellular compartment, we investigated whether this phenomenon was a peculiar feature of the malten chemical structure. To this end, plasmid DNA was exposed to compounds such as maltol, DMEDA and DMAMP, which represent part of the malten molecule and CDDP. Intriguingly, we found that the electrophoretic interferences on plasmid DNA are peculiar to the malten molecular structure and that CDDP, as expected (Bellon *et al*, 1991), causes just a slight retardation in the mobility of the supercoiled plasmid form (Figure 6D). Moreover, in accordance with cell-free studies, maltol, DMEDA and DMAMP failed to induce a biological response in any of the cellular models tested (data not shown).

Finally, we further investigated the possibility that malten could molecularly modify the DNA structure by assaying its interference on DNA amplification. Plasmid DNA was amplified after malten exposure, by Q-PCR, using three sets of primers sharing the same reverse oligonucleotide and able to generate amplicons of different sizes (121, 301 and 622 bp). First, we revealed that incubation with malten decreases the efficiency of PCR amplification (Figure 6E, upper panel). It is important that, despite doubling the amplicon size (121, 301 and 622 bp), malten induced an exponential loss of PCR efficiency (−7.13-fold, −99.7-fold, −6472-fold; Figure 6E upper panel—see Discussion).

Taken together, these results strongly support the hypothesis that malten can induce DNA structural modifications at a high level of complexity.

## Discussion

In this paper, we provide the biological properties of a new synthetic maltol-derived molecule (malten) and the preliminary attempt to understand its molecular mechanism of action.

The sensitivity to malten exposure of different tumour cell lines (derived from haematopoietic and solid tumours) was assayed in terms of decreased cell survival, programmed cell death induction and cell cycle perturbation.

Malten was able to drastically reduce the cell survival of all cell lines tested, showing interesting differences in terms of responsiveness and suggesting the presence of peculiar molecular properties that may render some cellular models more susceptible. High doses of malten activated the apoptotic cellular response, whereas sublethal treatments induced the accumulation of cells in both S and/or G2-M phases in almost all cell lines.

However, JURKAT and RH30 cells, two of the most sensitive cell lines in terms of reduced cell survival, showed a peculiar accumulation of cells in G2-M phases associated with a strong decrease of G1 phase, indicating that a possible different ability to activate cell cycle checkpoints may influence the antiproliferative effect of malten.

Gene expression studies, conducted at both mRNA and protein levels, support the biological data collected. In particular, upregulation of the broad-range cyclin-dependent kinase inhibitor (CDKi) p21, found in both JURKAT and U937 cells, is consistent with the late S and G2-M cell accumulation that we monitored.

However, the lack of p53 functionality in both cell lines (Cheng and Haas, 1990; Decker *et al*, 2003) indicates that the expression of p21 is induced in a p53-independent manner.

In addition to p21, we also found, in both JURKAT and U937 cells, upregulation of the INK4 family CDK-inhibitors p15 and p16, which are known to exert their activity during G1–S transition.

An increased expression of CUL3, a member of the cullin-RING ligase family with ubiquitinating ligase activity, was detected in JURKAT cells. Considering the crucial role of CUL3 protein in sustaining caspase-8 activity, and the robust increase of hypodiploidy observed in JURKAT cells exposed to malten, it is reasonable to hypothesise that CUL3 upregulation could contribute to the malten-induced apoptotic response (Békés and Salvesen, 2009; Lall, 2009).

The biological and molecular aspects monitored strongly indicate that malten exposure may activate a DNA damage response. The late S and G2-M block is usually associated with compounds known to form adducts with DNA and actually included in several therapeutic protocols (e.g., CDDP; Siddik, 2003).

In addition to p16 upregulation, which can also be induced as a consequence of DNA damage (Robles and Adami, 1998; Shapiro *et al*, 1998), malten treatments increased the expression of BRCA1, which is known to be involved in DNA-damage signalling through its multifunctional properties as controller of DNA replication, activator of G2-M checkpoint and as initiator of DNA repair processes (Huen *et al*, 2010).

Finally, the slight accumulation of p53 protein in JURKAT cells, although not functional, can be considered as a further phenomenon that supports the induction of a cellular response to DNA damage.

As a consequence of these observations, the possible DNA structural alterations induced by malten have been investigated by cell-free studies. We showed that malten exposure induces the formation of DNA molecules with strongly impaired electrophoretic mobility.

In addition, we showed that treatments of DNA with compounds that are part of the malten molecule did not show any DNA modification, indicating that changes in the electrophoretic properties of DNA are a peculiar feature of the malten molecule.

Interestingly, a similar interference with DNA electrophoretic mobility has been already described for some DNA alkylating compounds (Pereira *et al*, 1998; David-Cordonnier *et al*, 2002; Novakova *et al*, 2009) and has been associated with the formation of a DNA matrix of high molecular weight, which results from the interhelical crosslinking of multiple DNA molecules.

In linking cell-free assays with cellular response, it is extremely important to note that genomic DNA, extracted from malten-treated U937 cells, shows a dose-dependent reduction in its electrophoretic mobility. This finding suggests that the DNA-modifying function of malten could also occur in a cellular context.

Moreover, we accumulated further experimental observations suggesting that malten could trigger complex DNA structural modifications. The first indication is represented by the ability of malten to reduce the number of plasmid DNA available for amplification and thus decrease the efficiency of PCR assay (Figure 6, upper panel). Surprisingly, when amplicon length was increased (doubling the amplicon length from 121 to 622 bp), the number of amplifiable sequences decreases exponentially after incubation with malten (Figure 6E, upper panel).

In our opinion, these results are of great interest, especially considering the fact that the same assay shows a different behaviour if applied to plasmids exposed to CDDP. In fact, we reproduced a CDDP-dependent PCR inhibition similar to that observed by malten, considering the 121 bp amplicon (loss of amplificability of around seven- to eight-fold; Figure 6E, lower panel – see Supplementary Figure S3 for treatment of CDDP at the same concentration of malten). From this, we monitored a proportional decrease in PCR efficiency as the amplicon was progressively increased in length: CDDP modifies the single DNA molecules and is not able to generate intermolecular crosslinking (different DNA molecules covalently bound); thus, PCR inhibition is expected to be proportional to the increase in amplicon length. The observed exponential decreased amplificability of DNA induced by malten suggests the induction of DNA structural alterations at a higher level of complexity.

These observations, together with the evidence that malten-modified plasmid DNA is resistant to fragmentation by both sonication (Figure 6E) and nuclease digestion (see Supplementary Figure S4), whereas CDDP-treated DNA is fragmentable, strongly sustain the idea that malten could induce the formation of covalently bound DNA structures.

However, at this stage, other activities, such as the induction of ROS-mediated DNA damage already reported for higher doses of maltol with respect to those tested in our study (Murakami *et al*, 2006a, 2006b), cannot be completely excluded as part of the malten mechanism of action.

Although more studies will be necessary to completely understand the activity of malten, and the precise mechanism of action, we believe that this compound can be considered as an interesting molecular scaffold, worthy of further investigation, with the aim of developing new potential anticancer agents.

## Figures and Tables

**Table 1:** Cell-cycle modifications induced by malten treatments^a^

**Cell lines**	***μ* M**	**% G1**	**% S**	**% G2-M**
U937	0	39.3±3.5	51.3±5.5	6.9±0.9
	5	36.2±3.4	49.6±4.2	12.3±1.6^b^
	10	26.3±2.9^b^	59.1±5.3	13.7±1.9^b^
	25^c^	—	—	—
NB4	0	34.6±5.5	54.4±3.5	9.8±1.5
	5	34.1±2.6	52.2±3.3	11.6±2.4
	10	29.4±4.0	53.5±4.5	16.4±2.3^b^
	25^c^	—	—	—
JURKAT	0	40.8±3.7	34.1±5.3	22.5±2.5
	5	28.7±3.5^b^	37.1±3.7	30.5±2.9^b^
	10	19.1±4.6^b^	29.7±4.4	47.5±4.8^b^
	25^c^	—	—	—
HL-60	0	33.3±4.1	43.5±3.2	19.7±2.9
	5	29.3±2.4	48.2±4.4	20.1±2.8
	10	26.9±4.1	58.0±2.0^b^	14.9±0.4
	25	25.5±2.6	59.9±3.2^b^	13.8±1.4
U-373 MG	0	46.2±2.6	40.2±4.1	12.3±2.8
	5	40.8±3.0	43.1±3.6	15.6±3.1
	10	36.9±4.7	43.3±2.5	18.9±1.9^b^
	25	32.9±2.6^b^	46.7±4.0	20.8±2.0^b^
HeLa	0	59.6±3.3	29.1±2.0	10.1±1.7
	5	57.2±1.9	30.6±2.1	10.3±1.4
	10	56.4±2.1	28.4±2.1	13.6±2.1
	25	44.9±3.5^b^	40.0±2.8^b^	14.1±1.6^b^
H28	0	65.6±6.2	23.5±2.1	9.9±1.2
	5	58.7±3.0	28.6±3.7	10.1±1.0
	10	55.2±6.1	29.3±4.8	13.2±2.6
	25	51.2±2.6^b^	26.8±3.1	20.0±1.5^b^
RH30	0	34.4±3.2	44.7±5.0	20.0±2.1
	5	27.2±1.6^b^	38.7±1.8	33.8±1.5^b^
	10	15.3±2.7^b^	38.9±6.5	46.0±3.7^b^
	25	6.9±2.1^b^	40.2±2.0	52.0±3.6^b^

a Data represent mean values±standard deviation (s.d.) of three independent experiments.

b Statistically relevant variations respect to untreated controls (Student's *t*-test, *P*<0.05).

c Samples for which the software was not able to analyze the cell cycle.

## Supplementary Material

Supplementary Figure S1

Supplementary Figure S2A

Supplementary Figure S2B
