# Supplementary figures and images for: Malten, a new synthetic molecule showing in vitro antiproliferative activity against tumour cells and induction of complex DNA structural alterations
Source: Br J Cancer. 2010 Jun 22;103(2):239–48. doi: 10.1038/sj.bjc.6605745 (PMC2906739; doi:10.1038/sj.bjc.6605745)

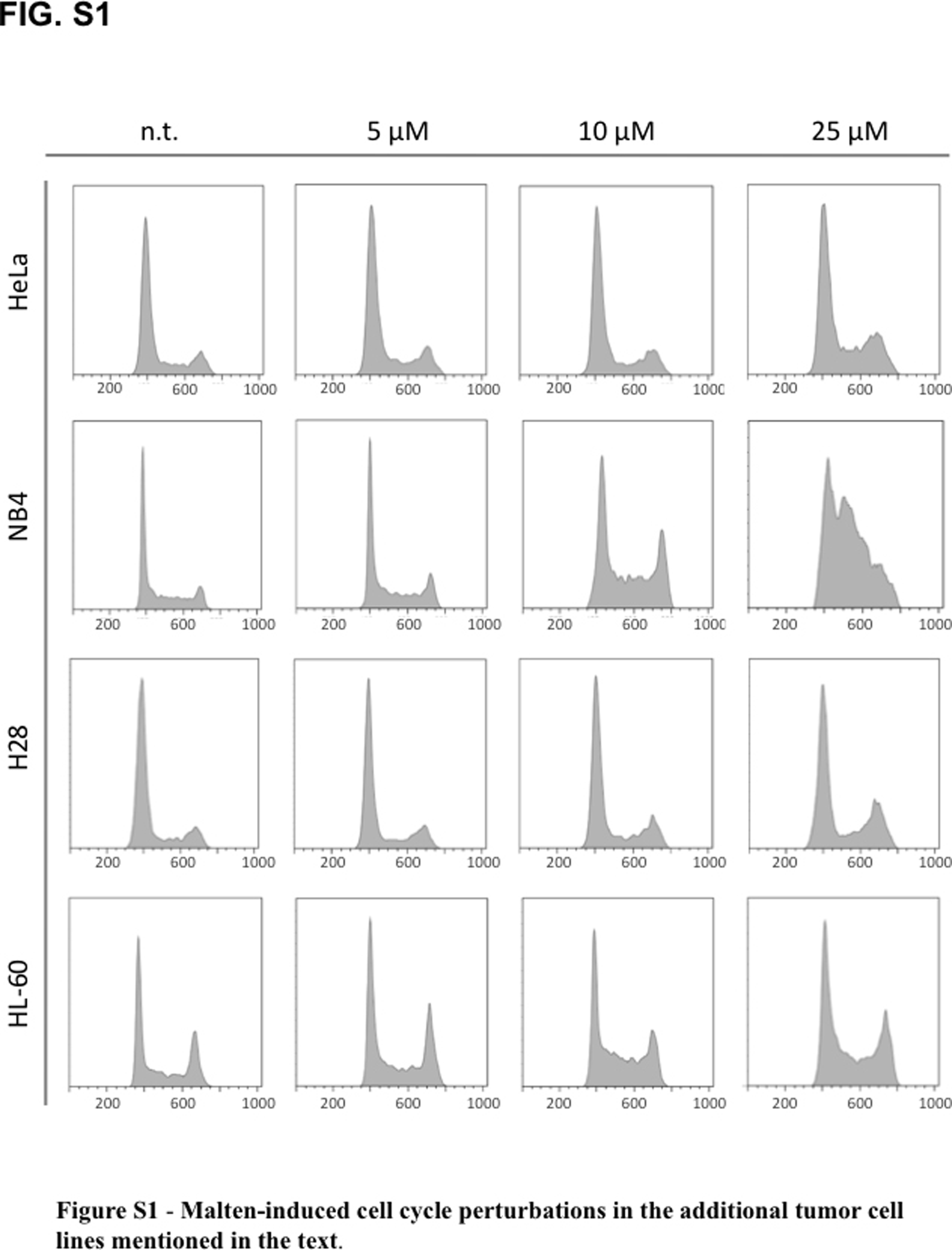

Supplement: Supplementary Figure S1 [file 6605745x1.tif]

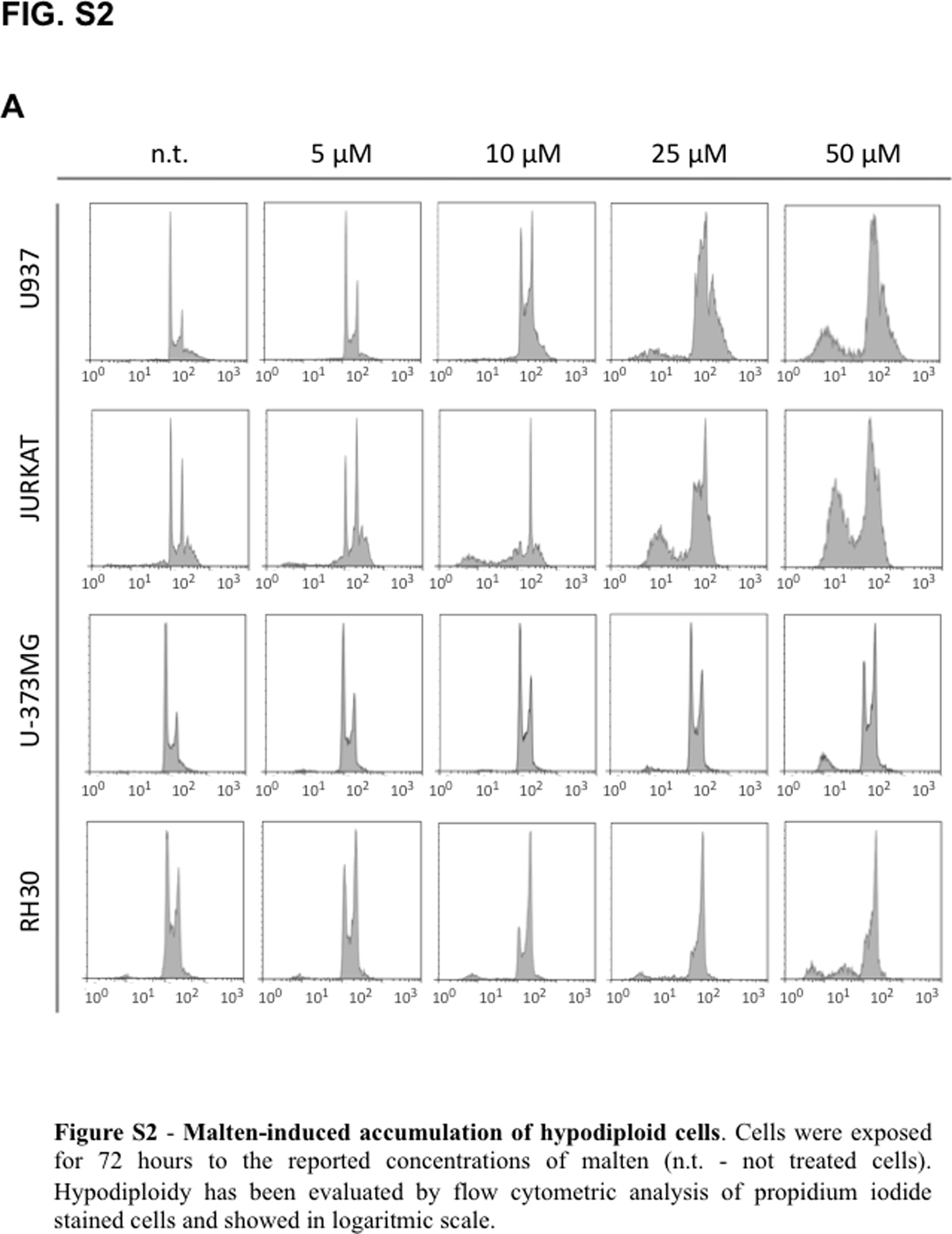

Supplement: Supplementary Figure S2A [file 6605745x2.tif]

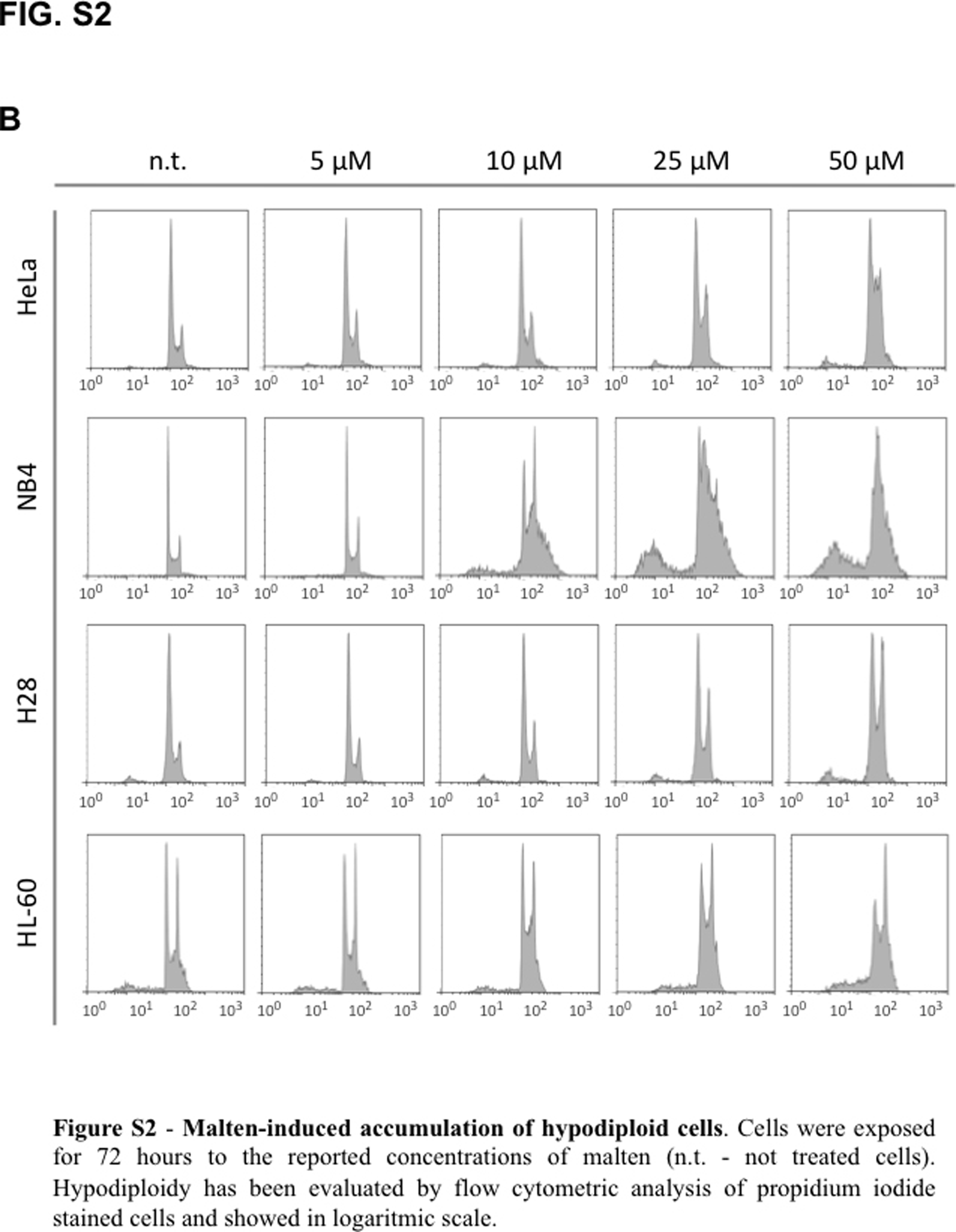

Supplement: Supplementary Figure S2B [file 6605745x3.tif]

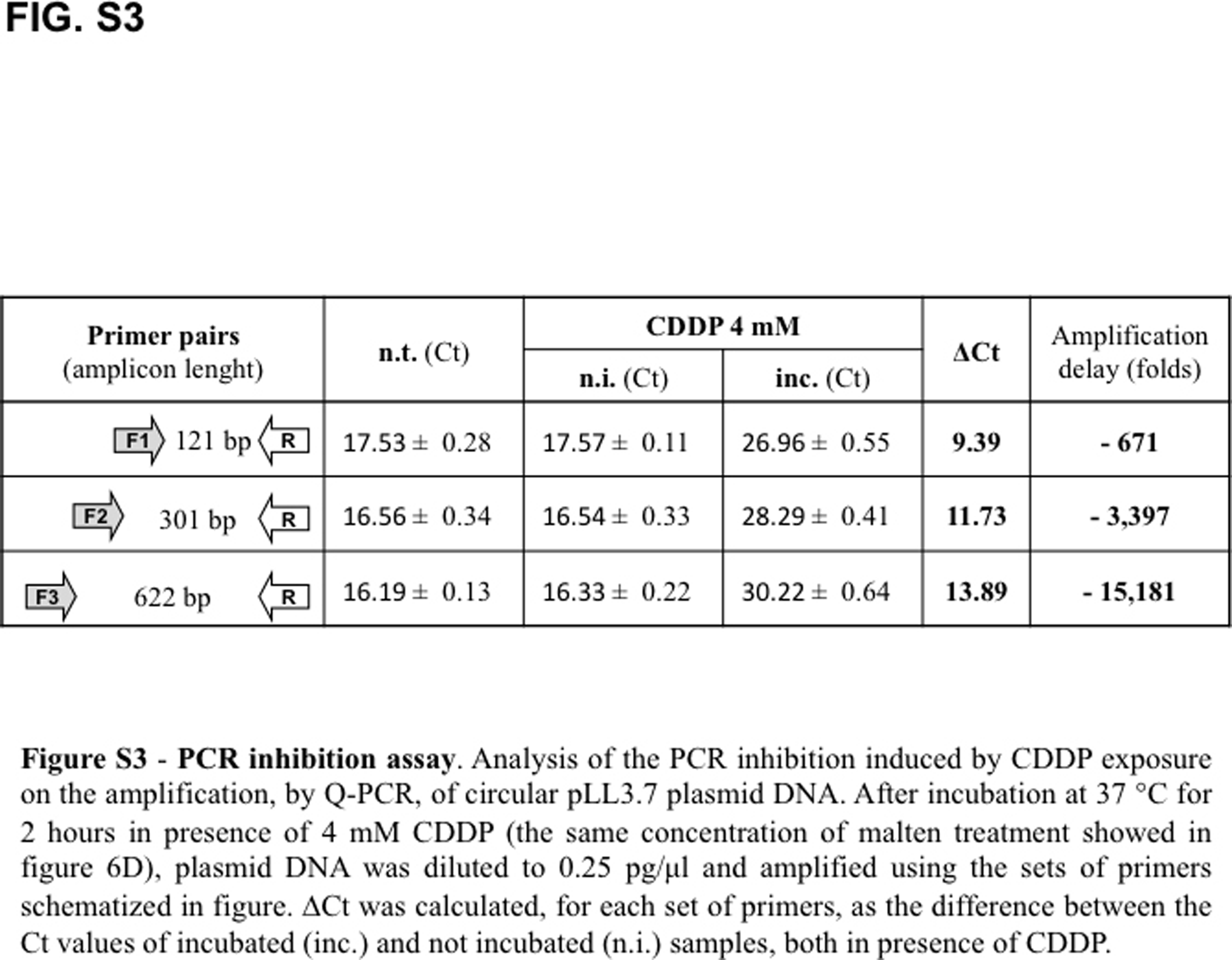

Supplement: Supplementary Figure S3 [file 6605745x4.tif]

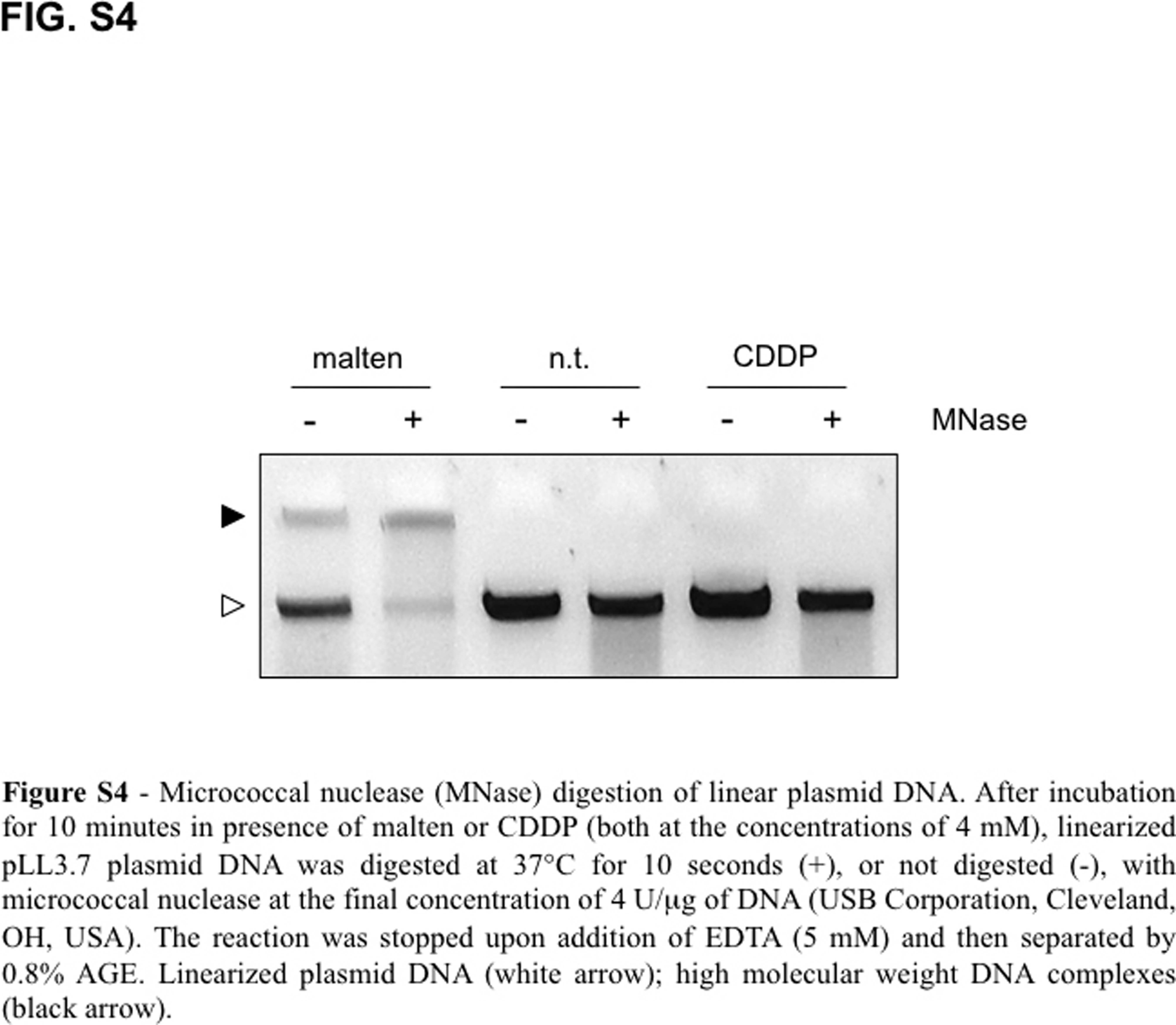

Supplement: Supplementary Figure S4 [file 6605745x5.tif]
